# 色谱-质谱技术在多发性硬化临床分析中的应用

**DOI:** 10.3724/SP.J.1123.2025.03024

**Published:** 2026-03-08

**Authors:** Zhenyu YAO, Yufei WANG, Xiaowen ZHU, Hong YANG, Jia WU, Guojun ZHANG

**Affiliations:** 1.首都医科大学附属北京天坛医院实验诊断中心，国家药监局体外诊断试剂质量控制重点实验室，北京市免疫试剂临床工程技术研究中心，首都医科大学神经感染实验诊断临床与基础联合实验室，北京 100070; 1. Laboratory Diagnosis Center，Beijing Tiantan Hospital，Capital Medical University，NMPA Key Laboratory for Quality Control for In Vitro Diagnostic，National Engineering Research Centre for Beijing Biochip Technology，Laboratory Diagnosis Platform for Nervous System Infectious Diseases，Laboratory for Clinical Medicine，Capital Medical University，Beijing 100070，China; 2.首都医科大学燕京医学院，北京 101300; 2. Yanjing Medical College，Capital Medical University，Beijing 101300，China

**Keywords:** 多发性硬化, 色谱-质谱, 生物标志物, 氨基酸代谢, 脂质代谢, multiple sclerosis （MuS）, chromatography-mass spectrometry, biomarkers, amino acid metabolism, lipid metabolism

## Abstract

多发性硬化（multiple sclerosis，MuS）是一种中枢神经系统慢性炎症和脱髓鞘疾病，全球患病率持续上升。其发病机制复杂，涉及免疫异常、遗传及环境因素，临床表现多样，在诊断与治疗方面面临挑战。代谢组学通过分析小分子代谢物，为揭示MuS病理机制和识别生物标志物开辟了新途径，尤其是在氨基酸和脂质代谢异常与炎症、髓鞘损伤及免疫反应关联的研究中发挥着重要作用。本文综述了色谱-质谱技术在MuS代谢标志物研究中的应用进展，聚焦氨基酸和脂质代谢的关键作用。回顾了液相色谱-串联质谱、高分辨质谱、气相色谱-质谱及基质辅助激光解吸电离-飞行时间质谱等技术，分析了MuS患者体液和组织样本中的代谢变化。通过总结代表性研究，探讨了这些技术在代谢物检测方面的应用。结果显示，MuS患者脑脊液中精氨酸代谢和色氨酸代谢途径紊乱，与氧化应激相关的焦谷氨酸水平发生变化；脂质代谢方面，脂肪酸、胆固醇和鞘脂代谢异常与免疫失调、髓鞘损伤及神经炎症关联。色谱-质谱技术以高灵敏度、高分辨率和高通量优势，为揭示MuS代谢异常发挥关键作用，为早期诊断、病情监测及个性化治疗提供了潜在标志物。

## Abstract (en)

Multiple sclerosis （MuS） is a chronic， inflammatory， demyelinating disease of the central nervous system， with a globally increasing prevalence. Its pathogenesis involves complex interactions among immune dysregulation， genetic predisposition， and environmental factors. These contribute to a wide spectrum of clinical manifestations， posing significant challenges for both diagnosis and treatment. Metabolomics， the comprehensive analysis of small-molecule metabolites， has emerged as a powerful approach to elucidate MuS pathophysiology and identify potential biomarkers. In particular， alterations in amino acid and lipid metabolism are closely associated with inflammation， myelin damage， neurotransmitter imbalance， and immune activation. This review summarizes recent advancements in the application of chromatography-mass spectrometry techniques to MuS biomarker research. Key technologies include liquid chromatography-tandem mass spectrometry （LC-MS/MS） for analyzing metabolites in biological fluids， high resolution mass spectrometry （HRMS） for precise metabolite identification， gas chromatography-mass spectrometry （GC-MS） for profiling volatile compounds， and matrix-assisted laser desorption ionization-time of flight mass spectrometry （MALDI-TOF MS） for mapping spatial metabolite distributions in tissues. Metabolomic analyses of MuS patient samples have revealed significant disruptions in amino acid metabolism. For instance， reduced arginine levels and elevated asymmetric dimethylarginine levels in cerebrospinal fluid suggest vascular dysfunction. Altered tryptophan metabolism， including altered levels of kynurenic acid and 5-hydroxytryptophan， reflects neurotransmitter imbalances. Increased pyroglutamic acid levels indicate oxidative stress. Lipid metabolism is also markedly perturbed in MuS. Changes in palmitic acid levels affect cell membrane integrity， while alterations in sphingolipids， key components of myelin， are linked to demyelination. Elevated oxysterols correlate with neuroinflammation and immune dysregulation. Additionally， systemic lipid changes， such as reduced low-density lipoprotein and altered high-density lipoprotein cholesterol levels， have been associated with disease activity and progression. Due to their high sensitivity， resolution， and throughput， chromatography-mass spectrometry techniques are well-suited for detecting these metabolic alterations. Representative studies demonstrate the utility of LC-MS/MS in detecting amino acid perturbations in plasma and cerebrospinal fluid （CSF）， GC-MS in identifying lipid abnormalities in serum， HRMS in generating detailed brain tissue metabolite profiles， and MALDI-TOF MS in visualizing lesion-specific metabolic distributions. These findings have led to the identification of promising biomarker candidates. For instance， decreased arginine may indicate endothelial dysfunction， while elevated oxysterols may serve as markers of neuroinflammatory activity. Such biomarkers hold potential for facilitating early diagnosis， disease monitoring， and personalized treatment strategies. Metabolomics via chromatography-mass spectrometry technologies has significantly advanced our understanding of MuS pathophysiology. Amino acid dysregulation affects neurotransmitter synthesis and immune modulation， while lipid imbalances impair myelin integrity and cellular signaling. These insights provide a foundation for the development of metabolite-based diagnostic tools and therapeutic interventions. Nonetheless， several challenges remain. MuS exhibits high heterogeneity， and metabolomic data are complex and variable. Improved biomarker specificity and standardization are needed for clinical application. Technological advances in chromatography-mass spectrometry resolution， along with standardized protocols， will be essential. Large-scale clinical validation across MuS subtypes is also critical. Integrating metabolomics with genomics and environmental factors may enhance biomarker reliability and elucidate individual disease trajectories. In conclusion， this review highlights the crucial role of chromatography-mass spectrometry-based metabolomics in MuS research. By unraveling the metabolic underpinnings of inflammation and demyelination， it provides valuable insights into disease mechanisms and introduces novel avenues for early diagnosis and targeted therapy. Collaborative research efforts and methodological rigor will be key to translating these discoveries into clinical impact.

多发性硬化（multiple sclerosis，MuS）是一种以中枢神经系统慢性炎症和脱髓鞘为特征的自身免疫性疾病，常导致神经功能障碍和临床残疾。根据最新研究，截至2023年，全球约有290万MuS患者，患病率持续上升^［1］^。根据病程特点，MuS通常被分为原发进展型（primary progressive，PP）、复发缓解型（relapsing remitting，RR）和继发进展型（secondary progressive，SP）。其发病机制涉及免疫系统异常、遗传因素和环境影响等多重因素，临床表现异质性强^［2］^。目前诊断MuS的方法包括临床诊断、脑脊液（cerebrospinal fluid，CSF）检查和磁共振成像（magnetic resonance imaging，MRI）检查。但仍有部分MuS患者的临床症状、影像学检查和实验室检查结果不符合典型MuS的诊断标准，此外MuS的一些诊断标准与视神经脊髓炎谱系疾病（neuromyelitis optica spectrum disorder，NMOSD）相符，并且MuS在少见人群（儿童、老年人和非白种人）中的诊断较为困难^［2］^。因此需要寻找更好的诊断方法或生物标志物来帮助临床对MuS的准确诊断以及分型，有助于辅助疾病的治疗策略和预后判断。

代谢组学是研究细胞代谢过程的底物、中间体或产物，可以提供生物体在特定时间点的生理快照，反映其健康状况、疾病状态或对环境刺激的响应。相比基因组学或蛋白质组学，代谢组学可以更直接地显示疾病的生理变化，并且代谢组学能够检测到临床症状出现前的代谢变化，这对MuS的早期诊断和分型尤为重要。色谱-质谱技术凭借其高灵敏度、高分辨率和高通量的优势，可以检测到微量或痕量的物质，这为研究MuS的代谢组学变化提供了重要工具。目前常用的色谱-质谱技术包括液相色谱-串联质谱（LC-MS/MS）、基质辅助激光解吸电离-飞行时间质谱（MALDI-TOF MS）、高分辨质谱（HRMS）、气相色谱-质谱（GC-MS）等。由于代谢物的类型和实验需求的不同，需要选择合适的技术进行分析。LC-MS/MS的优点在于适用范围广和样品无须衍生化处理，因此适用于某些极性和非极性化合物的分析，如脂质、氨基酸、多肽等。GC-MS优点在于分离效率高，具有良好的重现性和灵敏度，但不适合热不稳定物质和非挥发性化合物的分析。MALDI-TOF MS可对大分子代谢物进行快速、高灵敏度的分析，而HRMS适合小分子代谢物的研究。

研究表明，氨基酸代谢和脂质代谢的异常在MuS的炎症过程、神经递质调节、髓鞘损伤及免疫反应中发挥重要作用，为疾病的早期诊断、病情监测和治疗策略优化提供了重要线索^［3-5］^。本文旨在综述色谱-质谱技术在MuS代谢标志物研究中的应用进展，聚焦于氨基酸代谢和脂质代谢两个关键领域，通过总结这些研究成果，有望找到潜在的生物标志物。

## 1 色谱-质谱技术在MuS氨基酸代谢中的应用

近年来，色谱-质谱技术在MuS的研究使用中日益增多，可精确定量氨基酸、脂质等小分子，在MuS的研究中发挥着重要作用。

氨基酸在中枢神经系统和免疫系统中起重要作用，其既是蛋白质的原料，也是部分神经递质的前体，而神经递质在MuS的炎症过程和发病机制中起重要作用。L-精氨酸是一氧化氮（nitrogen monoxide，NO）的前体，可参与亚硝酸盐氧化物的代谢，产物为NO^［6］^。NO是一种由一氧化氮合成酶（nitric oxide synthetase，NOS）家族生成的自由基，包括神经元型NOS（nNOS）、内皮细胞型NOS（eNOS）和诱导型NOS（iNOS）。NO对MuS的影响较为复杂，其中由iNOS催化产生的大量NO可促进炎症反应和髓鞘损伤，而eNOS催化产生的NO则对MuS有保护作用^［7，8］^。有研究表明，在MuS活动性病变的位置发现NO浓度的增加^［9，10］^。这可能是由于iNOS催化L-精氨酸产生大量损伤性的NO。Židó等^［11］^研究采用HPLC-MS/MS对首次发作的MuS患者和健康对照者的CSF进行代谢组学分析，发现与对照组相比，首次发作的MuS患者CSF中的精氨酸水平显著降低。不对称二甲基精氨酸是精氨酸的代谢产物，它会竞争性抑制eNOS酶减少保护性NO的产生，常发挥调节血管功能的作用。Carlsson等^［12］^利用LC-MS/MS研究了12名SPMuS患者的氨基酸代谢变化，发现与健康对照组相比，SPMuS患者的不对称二甲基精氨酸水平显著升高（*P*<0.05）。综上，精氨酸代谢紊乱是影响MuS的重要途径，对精氨酸及其代谢物的研究有助于理解MuS发病机制和寻找相关生物标志物。

神经炎症通常由小胶质细胞和星形胶质细胞的激活引起，研究表明色氨酸代谢，尤其是犬尿氨酸途径可能在这一过程发挥重要作用^［13］^。犬尿氨酸途径大约占肝脏中色氨酸降解的90%，Bakker等^［14］^研究发现，低水平的色氨酸与神经退行性病变相关。Herman等^［15］^应用HRMS技术分析了30名RRMuS患者、16名SPMuS患者以及10名非炎症性神经系统疾病患者的CSF，发现与RRMuS和对照组相比，SPMuS患者色氨酸代谢改变更显著，其CSF中犬尿氨酸、5-羟色氨酸、*N*-乙酰血清素水平升高和5-羟基吲哚乙酸水平下降。Saraste等^［16］^直接探讨了小胶质细胞活化与血清犬尿氨酸途径代谢物之间的关联。研究通过采用UPLC-MS/MS技术和正电子发射断层扫描成像分析了MuS患者血清样本，发现血清中3-羟基犬尿氨酸水平降低与更高的小胶质细胞活化和疾病严重程度相关。此外，色氨酸代谢还与肠道微生物群和神经精神症状（如抑郁、疲劳）有关，这为未来的治疗提供了潜在方向^［17，18］^。但由于研究复杂性和个体差异，目前的证据仍需要进一步验证。

谷胱甘肽在抗氧化防御和细胞解毒中发挥重要作用。有研究发现，谷胱甘肽含量较高时能够帮助星形胶质细胞对进入大脑的自由基、过氧化物和其他化合物进行解毒^［19］^。焦谷氨酸是谷胱甘肽代谢途径中的一个中间产物，参与*γ*-谷氨酰循环，该循环负责谷胱甘肽的合成和分解。有研究显示，谷胱甘肽能够穿过血脑屏障，其代谢失衡可能造成MuS患者中枢神经系统中的氧化应激，这可能与MuS的炎症和神经退行性过程相关^［19，20］^。Poddighe等^［21］^通过GC-MS技术分析了MuS患者的血清，发现焦谷氨酸是区分MuS患者和健康对照的显著代谢物之一。Andersen等^［22］^研究分析了12名非吸烟、未接受药物治疗的男性MuS患者与13名年龄和身体质量指数匹配的健康男性对照的血清代谢物谱，通过非靶向二维GC-TOF MS，以及靶向的脂质与氨基酸定量分析筛选出12种与MuS显著相关的代谢物，其中焦谷氨酸显示出较高的诊断性能（AUC>80%），为MuS患者早期诊断和治疗靶点开发提供了关键线索。尽管上述研究提供了重要见解，但存在不同研究中焦谷氨酸水平的变化、入组的患者类型以及疾病阶段各异的问题。此外，焦谷氨酸的神经保护与神经毒性作用尚未完全阐明，未来的研究需要更系统地探索这些机制。

上述研究总结了精氨酸、色氨酸和焦谷氨酸代谢通路在MuS中的异常变化及其与神经炎症、氧化应激以及神经损伤的密切联系。总之，对这些通路的深度研究既反映了MuS的病理机制，还为寻找诊断MuS的生物标志物和开发新型治疗方法提供了思路。

## 2 色谱-质谱技术在MuS脂质代谢中的应用

脂质代谢包括脂肪酸、胆固醇和鞘脂的代谢过程，这些脂质在MuS的免疫和神经病理中发挥关键作用。研究显示，MuS患者免疫细胞中存在异常的脂质信号传导，这可能促进疾病的发生。激活的T细胞在MuS患者中显示出更高的细胞膜胆固醇和脂肪酸水平，这些变化影响了T细胞的功能和分化。此外，在MuS患者中观察到系统性脂质代谢紊乱，有研究发现低密度脂蛋白胆固醇（LDL）、高密度脂蛋白胆固醇（HDL）和载脂蛋白B（Apo-B）的水平变化与疾病活动和残疾程度密切相关^［23，24］^。Pineda-Torra等^［23］^研究发现在RRMuS患者中，升高的血清LDL水平与新的MRI病变相关，而更高的血清LDL和Apo-B水平则与更高的临床扩展致残量表（expanded disability status scale，EDSS）评分相关。Lorincz等^［24］^研究发现血清中高HDL水平与血脑屏障损伤的减少和脑脊液炎症浸润程度降低相关，HDL和Apo-AⅠ水平随时间增加可降低向PRMuS过渡的可能性以及脑萎缩的风险。在临床孤立综合征患者中，升高的Apo-B水平与EDSS评分相关。综上，血清中相关脂质水平的变化可用来预测MuS的进展程度及预后。

### 2.1 脂肪酸代谢

髓鞘主要由脂质组成，随着脱髓鞘过程的进展，脑脊液中脂肪酸水平增加。而髓鞘重建则会以脂肪酸作为基本原料，脂肪酸的水平随之降低。Ladakis等^［25］^通过GC-MS技术分析了慢性活动性或非活动性MuS患者病变边缘和核心，以及斑块周围白质样本与正常白质样本之间的代谢组差异。研究鉴定出783种代谢物，并发现与对照组相比，MuS病变白质中的长链脂肪酸与星形胶质细胞和免疫细胞丰度呈负相关，但与少突胶质细胞的丰度呈正相关。Židó等^［11］^研究采用HPLC-MS/MS方法对首次发作的MuS患者和健康对照者的CSF进行代谢组学分析，发现MuS患者在首次临床发作后的CSF中棕榈酸水平显著高于健康对照组（*P*=0.039）。这表明，棕榈酸可能与MuS的早期炎症活动和髓鞘损伤相关。棕榈酸作为髓鞘的基本组成成分，其水平的升高很可能反映了急性发作期间髓鞘的丢失和脂肪酸的释放。由于不同疾病阶段髓鞘的丢失与再生程度不同，棕榈酸的水平也因此发生变化。此外，有研究发现晚期MuS患者中的棕榈酸水平降低，这可能与脂肪酸在髓鞘修复过程中的消耗有关^［26，27］^。

综上，脂肪酸代谢在MuS的髓鞘损伤和修复过程中具有重要作用，MuS病变中脂肪酸与不同神经胶质细胞的丰度具有一定的相关性，且CSF中棕榈酸水平的变化可能反映髓鞘急性损伤和后期修复的动态过程。

### 2.2 胆固醇代谢

胆固醇是中枢神经系统中髓鞘形成的重要成分，研究表明其异常可能与MuS的病理过程有关^［23，28，29］^。氧化胆固醇是胆固醇代谢的重要产物，其水平变化与MuS中的神经退行性变化密切相关^［30］^。24*S*-羟基胆固醇是脑部特异性的胆固醇代谢产物，主要由脑部细胞合成并通过血脑屏障进入血液^［31］^。Teunissen等^［32］^采用GC-MS对60名MuS患者（20名RRMuS、20名SPMuS和20名PPMuS）和37名健康对照者进行分析，发现MuS患者（特别是PPMuS和年长型RRMuS）血清中24*S*-羟基胆固醇水平显著降低。7-酮胆固醇是通过非酶促氧化途径产生的胆固醇氧化产物，与氧化应激和神经损伤相关^［33，34］^。Mccomb等^［35］^采用LC-MS/MS技术分析了62名RRMuS患者和36名进行性MuS患者血清，发现MuS患者血清中7-酮胆固醇水平与神经轴突损伤标志物（如神经丝轻链）呈正相关，提示其可能参与神经退行性过程。并且，在随访研究中基线7-酮胆固醇水平与5年后的血清神经丝轻链水平显著相关，表明其有可能成为预测MuS患者神经轴突损伤的生物标志物。

MuS是一种以女性为主的疾病，女性患者中类固醇代谢的异常可能与疾病的发生和进展相关。Kanceva等^［36］^通过GC-MS技术分析了12名女性MuS患者和6名年龄性别匹配的健康对照者的血清类固醇水平，发现女性MuS患者血清中C21类固醇（皮质醇和孕酮）及其极性偶联物，以及C19类固醇（脱氢表雄酮）水平显著高于对照组。这一发现表明女性MuS患者可能存在特异性的类固醇代谢异常，这可能与疾病的炎症和免疫调节相关，但性别差异的研究仍需更多证明支持。

综上，胆固醇及其代谢物与MuS中的髓鞘形成、神经退行性变化及性别特异性炎症调节密切相关。氧化胆固醇的水平变化反映了MuS的神经损伤程度，其中7-酮胆固醇更作为潜在的神经退行性生物标志物展现出预测价值。女性MuS患者中类固醇代谢的异常反映了疾病的性别差异，为进一步探索MuS的病理机制提供了重要线索。因此开发能够对胆固醇及其代谢物进行精确检测的方法对MuS研究有着重要意义。Griffiths等^［37］^开发了一种结合电荷标记的HPLC-HRMS方法，用于分析人血清中的胆固醇及其代谢物。该方法能在17 min完成分析，灵敏度达到ng/mL水平，且样品制备简单高效，为进一步的临床研究和靶向代谢组学分析提供了强大工具。

### 2.3 鞘脂代谢

鞘脂在脑发育及髓鞘形成中起重要作用，特别是神经酰胺和鞘氨醇的水平变化，其代谢异常可能直接导致脱髓鞘。Dasgupta等^［38］^研究通过GC-MS和UPLC-MS/MS技术研究了大鼠脑发育过程中髓鞘的形成以及MuS患者脱髓鞘期间的鞘脂变化。研究发现，神经酰胺及其二氢形式的水平在胚胎期E期到出生后第21天（髓鞘形成高峰）期间逐步上升，随后下降并保持恒定比例（约4∶1~4.5∶1）。在MuS患者的大脑中，神经酰胺和鞘氨醇水平显著升高，可能导致少突胶质细胞死亡，进而引发脱髓鞘。这一发现表明，神经酰胺和鞘氨醇的代谢异常可能是MuS脱髓鞘的关键因素。Ladakis等^［25］^通过GC-MS技术比较了MuS患者慢性活动性或非活动性病变（边缘和核心以及斑块周围白质）与正常白质样本的代谢组差异，发现MuS病变白质中鞘氨醇、鞘磷脂和神经酰胺水平较高，而核苷酸、能量代谢物、溶血磷脂和单酰基甘油水平则较低。研究还发现，鞘脂和膜脂代谢物与星形胶质细胞和免疫细胞丰度呈正相关，内源性大麻素和单酰基甘油途径与星形胶质细胞和免疫细胞丰度呈负相关，但与少突胶质细胞的丰度呈正相关，这表明白质损伤导致的脂质代谢失调可能在MuS患者脱髓鞘过程中具有重要作用。

某些鞘脂组合可能帮助区分MuS与其他疾病。Shi等^［39］^采用LC-MS/MS和多重酶联免疫吸附测定技术分析CSF中脂质的代谢变化，发现CSF中3种鞘脂的组合在随机森林模型中AUC达到0.92，能够有效区分MuS和NMOSD，此结果不仅为MuS和NMOSD的鉴别提供了新思路，也强调了神经炎症和髓鞘分解在疾病中的关键作用。

其他鞘脂代谢产物可能也对MuS具有影响。鞘磷脂是髓鞘的主要成分，其代谢紊乱可能加剧少突胶质细胞凋亡和脱髓鞘^［40］^。Vergara等^［41］^采用非靶向脂质组学和MALDI-TOF MS技术（以9-氨基吖啶为基质，负离子模式）分析MuS患者和健康对照组的CD4^+^T淋巴细胞。研究结果发现，MuS患者CD4^+^T淋巴细胞中磷脂酰胆碱和鞘磷脂等代谢物丰度显著增加，这些变化可能调节MuS的免疫反应，提示淋巴细胞中脂质代谢改变可能是MuS特异性的潜在生物标志物，同时验证了MALDI-TOF MS技术在脂质分析中的适用性。Carlsson等^［12］^利用LC-MS/MS研究了12名SPMuS患者的脂质代谢变化，发现与健康对照组相比，SPMuS患者的甘油磷脂PC-O（34∶0）水平显著升高（*P*<0.05）。这一发现进一步支持了鞘脂代谢异常在MuS进展中的作用。Niehaus等^［42］^利用MALDI-TOF MS和激光烧灼-电感耦合等离子体质谱（LA-ICP MS）技术发现MuS病变区域的脂质分布异常，部分脂质（磷脂酰胆碱、鞘磷脂）显著减少，为MuS的病理机制研究和诊断靶点开发提供了新视角。

综上所述，鞘脂在MuS的病理过程中发挥重要作用，特别是神经酰胺、鞘氨醇和鞘磷脂等代谢产物的水平变化与脱髓鞘、神经炎症和轴突损伤密切相关。其代谢异常不仅反映了其作为疾病生物标志物的潜力，还有可能作为后续治疗的靶点。高效的分析方法为这些代谢物的检测提供了强大工具，但仍需要更多深入的研究来确认这些生物标志物的临床应用价值。表1总结了部分具有代表性的研究成果，显示多种涉及氨基酸和脂质代谢的小分子可能与MuS的发病机制相关。

**表1:** 与多发性硬化相关的小分子代谢标志物

Sample	Technologies	Metabolisms	Dysregulation	Ref.
Cerebrospinal fluid （CSF）	HPLC-MS/MS	arginine	↓	［^11^］
		palmitic acid	↓	
Serum	LC-MS/MS	asymmetric dimethylarginine	↑	［^12^］
		glycerophospholipid PC-O （34∶0）	↑	
CSF	HRMS	kynurenine	↑	［^15^］
		5-hydroxytryptophan	↑	
		*N*-acetylserotonin	↑	
		5-hydroxyindoleacetic acid	↓	
Serum	UPLC-MS/MS	3-hydroxykynurenine	↓	［^16^］
Serum	GC-MS	pyroglutamate	↓	［^21^］
Serum	non-targeted two-dimensional GC-TOF MS	pyroglutamate	↑	［^22^］
White matter	GC-MS	long-chain fatty acids	negatively correlated with astrocyte abundance， positively correlated with oligodendrocyte abundance	［^25^］
Serum	GC-MS	24*S*-hydroxycholesterol	↓	［^32^］
Serum	LC-MS/MS	7-ketocholesterol	positively correlated with neurofilament light chains	［^35^］
Serum	GC-MS	C21 steroids （cortisol and progesterone） and their polar conjugates	↑	［^36^］
		C19 steroid （dehydroepiandrosterone）	↑	
Rat brain	GC-MS， UPLC-MS/MS	ceramides and their dihydro forms	↑	［^38^］
Brain	GC-MS UPLC-MS/MS	ceramides， sphingosine	↑	
CSF	LC-MS/MS	three types of sphingolipids	AUC>0.92	［^39^］
CD4^+^T cell	MALDI-TOF MS	phosphatidylcholine， sphingomyelin	↑	［^41^］
Brain	MALDI-TOF MS， LA-ICP MS	phosphatidylcholine， sphingomyelin	↓	［^42^］

LA-ICP-MS： laser ablation-inductively coupled plasma mass spectrometry； ↑： increase； ↓： decrease.

此外，能量代谢也在MuS代谢组学中得到了关注。能量代谢包括分解代谢和合成代谢，主要依赖线粒体功能、代谢中间产物和关键酶的活动。过去有研究在MuS患者中观察到了生物素、铁、维生素D（VitD）、葡萄糖以及线粒体代谢途径的异常，说明能量代谢可能与MuS的病理过程相关，但仍需要更多的研究确认^［43］^。Qu等^［44］^研究开发了一种新型LC-MS/MS方法，结合捕获微液相色谱-串联窄窗隔离选择反应监测质谱，成功实现了对VitD代谢物的超灵敏、稳定和高通量定量分析。该方法应用于218名MuS患者血清VitD代谢物的检测，结果显示，该方法的定量限明显低于现有的LC-MS/MS方法。Tavazzi等^［45］^使用HPLC方法分析了170例MuS患者和163名健康对照者的血清，结果发现，与对照组相比，MuS患者表现出多种代谢物的增加，包括尿酸、肌酐、次黄嘌呤、黄嘌呤、尿苷、*β*-假尿苷、丙二醛、亚硝酸盐和硝酸盐，以及抗坏血酸的减少。此外，Poddighe等^［21］^使用GC-MS技术分析了MuS患者与健康对照者的血浆。研究发现两组之间磷酸盐、果糖和肌醇水平发生变化。综上所述，能量代谢途径中的多种代谢物在MuS中表现出异常变化，这些变化涉及能量产生、氧化应激和神经修复等机制。尽管当前研究已取得一定进展，但有关色谱-质谱技术在能量代谢研究中的应用较少，未来需要对能量代谢开发新的技术，以推动有关MuS能量代谢机制研究和寻找生物标志物的研究。

## 3 总结与讨论

色谱-质谱技术在MuS代谢标志物研究中展现了重要价值，尤其是在揭示氨基酸和脂质代谢异常方面。MuS患者氨基酸代谢存在显著改变，例如精氨酸和色氨酸代谢途径的紊乱，这些变化与炎症反应和神经损伤密切相关。氧化应激相关代谢物如焦谷氨酸的异常，则进一步强调了氧化应激在MuS中的重要性。在脂质代谢方面，MuS患者表现出胆固醇、鞘磷脂及脂肪酸等代谢的显著异常，这些改变与免疫功能失调、髓鞘损伤和神经炎症密切相关。CSF和血清中棕榈酸、鞘脂及氧化胆固醇水平的波动，不仅反映了脱髓鞘和髓鞘修复的动态过程，还可能用于区分MuS与其他神经免疫疾病。此外，系统性脂质代谢指标（如LDL和HDL水平）的变化与疾病活动性相关，为监测疾病进展提供了线索。

色谱-质谱技术在MuS代谢研究中的应用前景广阔，但存在一些局限性。首先，样本选择（如CSF或血清）对代谢物检测的准确性影响较大，目前尚不清楚某些代谢物是否能够通过血脑屏障，这就无法验证CSF或大脑中的浓度是否会受到外周血中浓度的影响。其次，MuS的异质性（如疾病亚型和疾病进展）可能导致代谢特征不一致，并且一些代谢物可能受进食与空腹状态、药物、一天中采集血液的时间以及处理时间等多种因素的影响。此外，研究的样本量较小，这也可能导致研究结果出现偏倚。未来研究应该扩大样本量和增加多中心的研究，并对代谢物在外周血中的浓度和CSF中浓度的关系进行探究。另外，对MuS患者的疾病亚型和入组标准应加以明确和优化，以提高研究结论的准确性。

未来的研究方向可围绕技术进步、研究方法的完善和临床应用的拓展展开。可结合多组学技术，利用色谱-质谱技术检测代谢物，并与其他组学数据联合分析，挖掘关键标志物和代谢途径。另外，可以长期随访MuS患者，研究其代谢变化与疾病进展的关系。还可以通过色谱-质谱技术分析患者的代谢谱，结合临床数据筛选适合个体化治疗的靶点和药物。最后，针对色谱-质谱技术本身可以进行一些优化和改进，例如优化色谱分离条件，提升质谱分辨率和开发新型离子化技术来提高灵敏度。

综上所述，色谱-质谱技术在MuS代谢标志物研究中已取得初步成效，揭示了氨基酸和脂质代谢异常的关键作用。未来通过技术改进和临床验证，筛选出的代谢标志物有望为MuS的诊断、监测和个性化治疗提供重要支持。

## References

[1] Koskie B . Multiple Sclerosis： Facts， Statistics， and You. （2025-01-29） ［2025-05-15］. https://www.healthline.com/health/multiple-sclerosis/facts-statistics-infographichttps://www.healthline.com/health/multiple-sclerosis/facts-statistics-infographic

[2] Zhong X N， Hu X Q . Chinese Journal of Neuroimmunology and Neurology， 2019， 26（2）： 80

[3] Hu J， Melchor G S， Ladakis D， et al . Regen Med， 2024， 9： 1 10.1038/s41536-023-00345-9 PMC10762216 38167866

[4] Broos J Y， van der Burgt R T M， Konings J， et al . J Neuroinflammation， 2024， 21（21）： 1 38233951 10.1186/s12974-023-02981-w PMC10792915

[5] Zahoor I， Rui B， Khan J， et al . Cell Mol Life Sci， 2021， 78（7）： 3181 33449145 10.1007/s00018-020-03733-2 PMC8038957

[6] Sylvestre D A， Slupsky C M， Aviv R I， et al . Brain Res， 2020， 1732： 146589 31816317 10.1016/j.brainres.2019.146589

[7] Villadangos L， Serrador J M . Mol Sci， 2024， 25（24）： 13402 10.3390/ijms252413402 PMC11678294 39769167

[8] Shi B K， Li S L . The Journal of Medical Theory and Practice， 2020， 33（24）： 4081

[9] Smith K J， Lassmann H . BMC Neurol， 2002， 1（4）： 232 10.1016/s1474-4422(02)00102-3 12849456

[10] Encinas J M， Manganas L， Enikolopov G， et al . Curr Neurol Neurosci Rep， 2005， 5（3）： 232 15865889 10.1007/s11910-005-0051-y

[11] Židó M， Kačer D， Valeš K， et al . Front Neurol， 2022， 13： 874121 35693010 10.3389/fneur.2022.874121 PMC9178205

[12] Carlsson H， Abujrais S， Herman S， et al . Metabolomics， 2020， 16（2）： 26 32052189 10.1007/s11306-020-1648-5 PMC7015966

[13] Dobson R， Giovannoni G . Eur J Neurol， 2019， 26： 27 30300457 10.1111/ene.13819

[14] Bakker L， Ramakers I H， Greevenbroek M M J， et al . J Neurol Sci， 2025， 474： 123522 40367835 10.1016/j.jns.2025.123522

[15] Herman S， Åkerfeldt T， Spjuth O， et al . Cells， 2019， 8（2）： 84 30678351 10.3390/cells8020084 PMC6406712

[16] Saraste M， Matilainen M， Rajda C， et al . Mult Scler， 2022， 59： 103667 10.1016/j.msard.2022.103667 35151985

[17] Tan L S Y， Francis H M， Lim C K . Brain Behav Immun， 2021， 12： 100201 10.1016/j.bbih.2021.100201 PMC8474511 34589733

[18] Gaetani L， Boscaro F， Pieraccini G， et al . Front Immunol， 2020， 11： 157 32132996 10.3389/fimmu.2020.00157 PMC7041364

[19] Dringen R， Arend C . Neurochem， 2025， 169（5）： e70073 10.1111/jnc.70073 PMC12046376 40313177

[20] Pederzolli C D， Mescka C P， Zandona B R， et al . Metab Brain Dis， 2010， 25： 145 20431931 10.1007/s11011-010-9190-1

[21] Poddighe S， Murgia F， Lorefice L， et al . Int J Biochem Cell Biol， 2017， 93： 148 28720279 10.1016/j.biocel.2017.07.004

[22] Andersen S L， Briggs F B S， Winnike J H， et al . Mult Scler， 2019， 31： 12 10.1016/j.msard.2019.03.006 PMC6548586 30877925

[23] Pineda-Torra I， Siddique S， Waddington K E， et al . Front Endocrinol （Lausanne）， 2021， 12： 639757 33927692 10.3389/fendo.2021.639757 PMC8076792

[24] Lorincz B， Jury E C， Vrablik M， et al . Autoimmun Rev， 2022， 21（6）： 103088 35398271 10.1016/j.autrev.2022.103088

[25] Ladakis D C， Pedrini E， Maria I， et al . Neurol Neuroimmun， 2024， 11： e200219 10.1212/NXI.0000000000200219 PMC11073865 38547430

[26] Gonzalo H， Brieva L， Tatzber F， et al . Neurochem， 2012， 123： 622 10.1111/j.1471-4159.2012.07934.x 22924648

[27] Oliveira E M L， Montani D A， Oliveira-Silva D， et al . Arq Neuropsiquiatr， 2019， 77： 696 31664345 10.1590/0004-282X20190122

[28] Zhornitsky S， McKay K A， MetzL M， et al . Mult Scler， 2016， 5： 53 10.1016/j.msard.2015.10.005 26856944

[29] Vejux A， Ghzaiel I， Nury T， et al . J Steroid Biochem Mol Biol， 2021， 210： 105870 33684483 10.1016/j.jsbmb.2021.105870

[30] Hasegawa S， Watanabe S， Fujimoto S， et al . Neurosci Res， 2024， 208： 29 38897234 10.1016/j.neures.2024.06.005

[31] Wheless J W . J Epilepsy， 2025， 66（1）： 33 10.1111/epi.18174 PMC11742637 39487852

[32] Teunissen C E， Dijkstra C D， Polman C H， et al . Neurosci Lett， 2003， 347（3）： 159 12875910 10.1016/s0304-3940(03)00667-0

[33] Duc D， Vigne S， Pot C . Int J Mol Sci， 2019， 20（18）： 4522 31547302 10.3390/ijms20184522 PMC6770630

[34] Testa G， Staurenghi E， Zerbinati C， et al . Redox Biol， 2016， 10： 24 27687218 10.1016/j.redox.2016.09.001 PMC5040635

[35] McComb M， Browne R W， Bhattacharya S， et al . Mult Scler， 2021， 50： 102864 10.1016/j.msard.2021.102864 33677412

[36] Kanceva R， Stárka L， Kancheva L， et al . Physiol Res， 2015， 64（2）： S247 26680486 10.33549/physiolres.933145

[37] Griffiths W J， Hornshaw M， Woffendin G， et al . Proteome Res， 2008， 7（8）： 3602 10.1021/pr8001639 PMC2567817 18605750

[38] Dasgupta S， Ray S K . J Neurol Psychol， 2017， 5（1）： 10 10.13188/2332-3469.1000035 PMC6190913 30338269

[39] Shi L， Ghezzi L， Fenoglio C， et al . J Neurol Neurosurg Psychiatry， 2024， 96（1）： 54 38844340 10.1136/jnnp-2024-333774 PMC11672031

[40] Jana A， Pahan K . Neuromolecular Med， 2010， 12（4）： 351 20607622 10.1007/s12017-010-8128-4 PMC2987401

[41] Vergara D， D′Alessandro M， Rizzello A， et al . BMC Neurosci， 2015， 16： 46 26205308 10.1186/s12868-015-0183-1 PMC4513631

[42] Niehaus P， Gonzalez de Vega R， Haindl M T， et al . Talanta， 2024， 270： 125518 38128277 10.1016/j.talanta.2023.125518

[43] Heidker R M， Emerson M R， LeVine S M . Neural Regen Res， 2017， 12（8）： 1262 28966637 10.4103/1673-5374.213542 PMC5607817

[44] Qu F， Zhang M， Weinstock-Guttman B， et al . Sci Rep， 2024， 14（1）： 5545 38448553 10.1038/s41598-024-55939-0 PMC10918069

[45] Tavazzi B， Batocchi A P， Amorini A M， et al . Mult Scler J， 2011： 167156 10.1155/2011/167156 PMC3196932 22096628

